# Distinct peripheral B-cell immunophenotypes define clinical heterogeneity in childhood-onset systemic lupus erythematosus

**DOI:** 10.1007/s12519-026-01082-x

**Published:** 2026-09-01

**Authors:** Li-Yun Xu, Hui Chen, Yong-Zhen Li, Lan Luo, Ming Yang, Xiao-Chuan Wu

**Affiliations:** 1 Department of Pediatrics, The Second Xiangya Hospital of Central South University, 135 Renmin Road, Furong District, Changsha 410000, China; 2 Department of Pediatrics, Hunan Children’s Hospital, Changsha, China; 3 Department of Dermatology, The Second Xiangya Hospital of Central South University, Changsha, China; 4 Hunan Key Laboratory of Medical Epigenomics, Changsha, China

**Keywords:** B-lymphocytes, Biomarkers, Childhood-onset systemic lupus erythematosus, Disease activity, Plasma cells

## Abstract

**Background:**

B-cell dysregulation is central to the pathogenesis of childhood-onset systemic lupus erythematosus (cSLE), yet the B-cell immunophenotypes across disease states remain incompletely defined. This study aimed to investigate the mechanistic basis and clinical relevance of B-cell–mediated immune dysregulation in cSLE.

**Methods:**

Peripheral blood samples from 64 cSLE patients and 40 age- and sex-matched healthy controls (HCs) were analyzed using flow cytometry. The subsets were compared across disease states, including lupus low disease activity state (LLDAS), active disease, and clinical flare. Diagnostic performance was evaluated using logistic regression and receiver operating characteristic analyses.

**Results:**

Compared with HCs, patients with cSLE exhibited reduced unswitched memory B cells and expanded pathogenic subsets, including double-negative (DN) B cells, age-associated B cells (ABCs), short-lived plasma cells (SLPCs), and long-lived plasma cells (LLPCs) (all *P* < 0.05). LLPCs showed the strongest disease association [area under the curve (AUC) = 0.855] and a combined model further improved discrimination (AUC = 0.936). Active disease was characterized by expanded DN B cells, ABCs, SLPCs, and LLPCs (all *P* < 0.05). Clinical flare involved selective enrichment of ABCs, DN B cells, and plasma cells without further increase in total B cells. Of note, LLPCs remained elevated in LLDAS compared with HCs (*P* < 0.01). ABCs were negatively correlated with complement C3 levels (*r* =  − 0.26, *P* = 0.04).

**Conclusions:**

cSLE is characterized by distinct peripheral B-cell immunophenotypes across different clinical states, marked by expansion of ABCs and plasma-cell populations. Persistent LLPC enrichment during clinical quiescence suggests incomplete immunological restoration and may contribute to disease heterogeneity and relapse susceptibility.

**Graphical abstract:**

**Figure:**
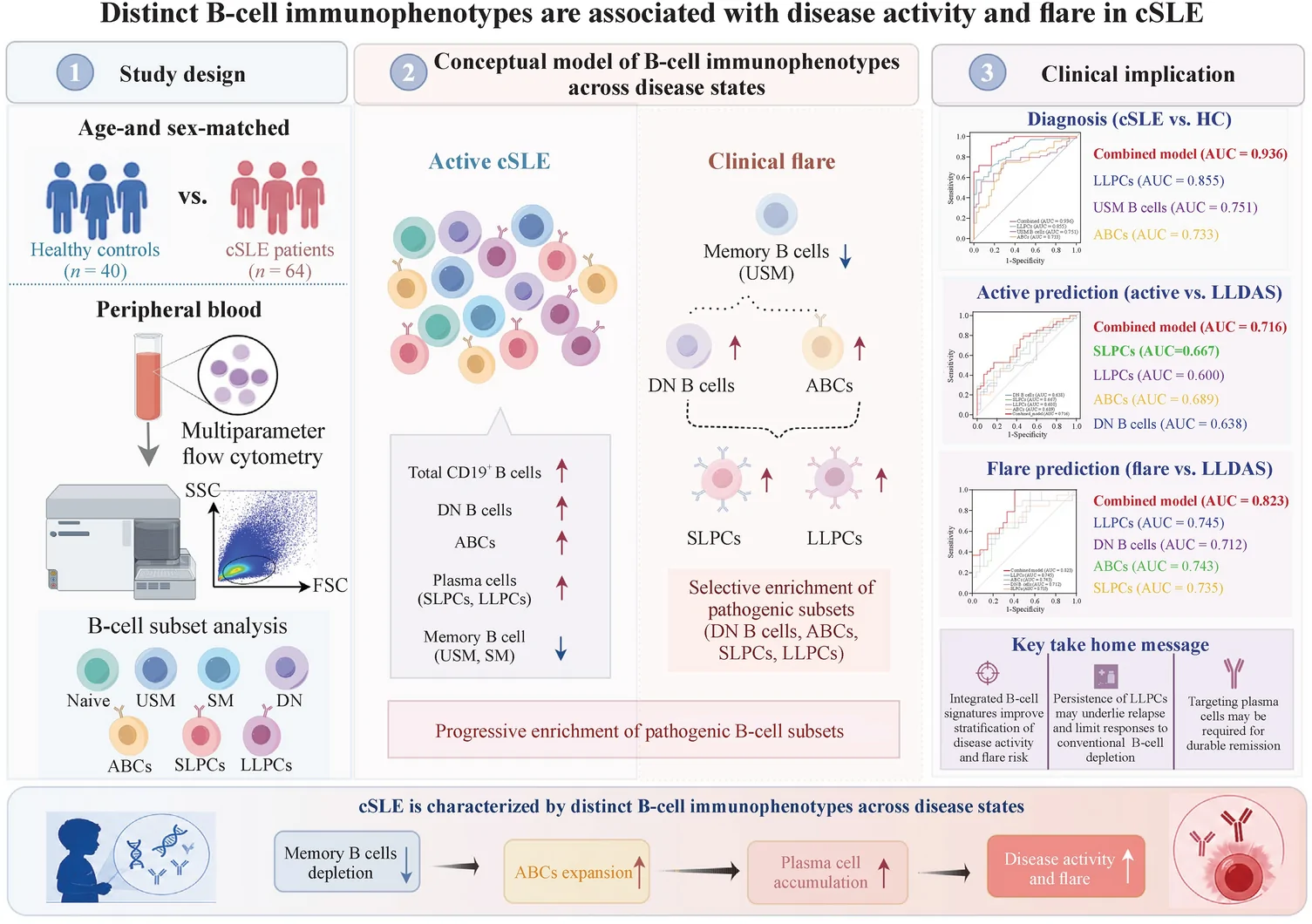

**Supplementary Information:**

The online version contains supplementary material available at https://doi.org/10.1007/s12519-026-01082-x.

## Introduction

Childhood-onset systemic lupus erythematosus (cSLE) is a severe multisystem autoimmune disease characterized by profound immune dysregulation and the production of diverse autoantibodies [1, 2]. Compared with adult-onset disease, cSLE is typically associated with higher disease activity, more severe organ involvement, and poorer long-term outcomes [3, 4]. Despite advances in immunosuppressive therapy, disease monitoring and prediction of relapse remain major clinical challenges, underscoring the need for a deeper understanding of its immunopathogenesis.

B-cell dysregulation is a central feature of SLE pathogenesis, extending beyond autoantibody production to include antigen presentation and cytokine secretion [5, 6]. Advanced immunophenotyping has identified multiple aberrant B-cell subsets in adult SLE, including double-negative (DN) B cells, age-associated B cells (ABCs), and antibody-secreting cells (ASCs), reflecting dysregulated germinal center (GC) and extrafollicular (EF) responses [7–9]. Among DN B cells, the EF DN2 subset has been implicated in pathogenic autoreactive responses, whereas ABCs represent a chronically activated B-cell subset enriched in autoimmune diseases and capable of differentiating into ASCs [8,^10^]. Among ASCs, the short-lived plasma cells (SLPCs) are rapidly generated effector cells associated with active immune responses, while long-lived plasma cells (LLPCs) can survive for prolonged periods in specialized niches and sustain autoantibody production independently of continuous antigen stimulation [11, 12]. However, these findings are largely derived from adult cohorts and whether similar or distinct patterns exist in cSLE remains incompletely defined.

Importantly, most existing studies rely on static comparisons between patients and healthy controls (HCs), providing limited insight into how B-cell subsets evolve across disease states. In particular, it remains unclear whether disease activity and clinical flare represent distinct immunological programs or simply different points along a continuum. This knowledge gap hampers the identification of early warning signals for disease instability and limits the development of precision monitoring strategies in cSLE.

Although B-cell–targeted therapies, including anti-CD20 monoclonal antibodies and B-cell activating factor (BAFF) inhibitors, have improved disease management, treatment responses remain variable and often incomplete [13]. Of note, the limited efficacy of anti-CD20 therapies in clinical trials suggests that simple B-cell depletion may be insufficient to achieve sustained remission [14, 15]. Emerging evidence indicates that LLPCs, which lack CD19 and CD20 expression, may persist despite therapy and continue to drive autoantibody production.

In this study, we performed comprehensive immunophenotyping of peripheral B-cell subsets in patients with cSLE using multiparameter flow cytometry. We sought to define the overall architecture of peripheral B-cell differentiation, characterize distinct B-cell immunophenotypes associated with disease activity and clinical flare, and evaluate the diagnostic utility of integrated B-cell signatures. By characterizing B-cell immunophenotypes across different clinical states, this study aims to provide mechanistic and clinically relevant insights into B-cell–mediated immune dysregulation in cSLE.

## Methods

### Study design and participants

This prospective, cross-sectional observational study was conducted to systematically characterize both the quantitative and phenotypic remodeling of peripheral B-cell subsets in patients with cSLE compared with age- and sex-matched HCs. The study protocol was approved by the Ethics Committee of the Second Xiangya Hospital of Central South University (Changsha, China) (LYG20220035). Informed consent was obtained from all research individuals (or their parents/legal guardians).

Eligible patients met the following criteria: diagnosis of cSLE according to the 2012 Systemic Lupus International Collaborating Clinics classification criteria and/or the 2019 European League Against Rheumatism/American College of Rheumatology classification criteria [16, 17] and age between 4 and 17 years at enrollment. The exclusion criteria included coexisting autoimmune diseases, hematological malignancies, or primary immunodeficiencies. Acute or chronic infections at the time of sampling. Prior receipt of any B-cell-targeted therapies, including anti-CD20 monoclonal antibodies (e.g., rituximab) or BAFF inhibitors (e.g., belimumab). Receipt of blood product transfusion within the preceding six months.

Disease activity was assessed using the SLE disease activity index 2000 (SLEDAI-2K) [18]. The patients were stratified into active disease and lupus low disease activity state (LLDAS) according to established definitions [19]. Active disease was defined as a SLEDAI-2K score ≥ 6, consistent with moderate-to-high disease activity. Within the active group, a subset of patients was further classified as experiencing clinical flare based on physician assessment and standardized criteria, including worsening clinical manifestations and/or escalation of immunosuppressive therapy. Age- and sex-matched healthy children undergoing routine health examinations were recruited as HCs. Children with a history of autoimmune, inflammatory, allergic, hematologic, or malignant diseases, acute or chronic infections, immunomodulatory medication use, or abnormal routine laboratory findings were excluded.

### Data collection

Demographic data (age and sex), clinical characteristics (disease duration, organ involvement, disease activity scores, and medication exposure), and laboratory parameters (complement levels, autoantibody profiles, and inflammatory markers) were extracted from electronic medical records using standardized case report forms. All data were independently reviewed and cross-validated by two investigators to ensure accuracy and consistency.

### Flow cytometric immunophenotyping of B-cell subsets

Peripheral blood samples were collected into EDTA-anticoagulated tubes and processed within four hours. Peripheral blood mononuclear cells (PBMCs) were isolated via density-gradient centrifugation using human lymphocyte separation medium (TBD Science, Tianjin, China). Following red blood cell lysis and washing with phosphate-buffered saline, the cells were resuspended at a concentration of 1 × 10^6^ cells/mL. Freshly isolated PBMCs were immediately subjected to antibody staining and flow cytometric analysis.

**Fig. 1:**
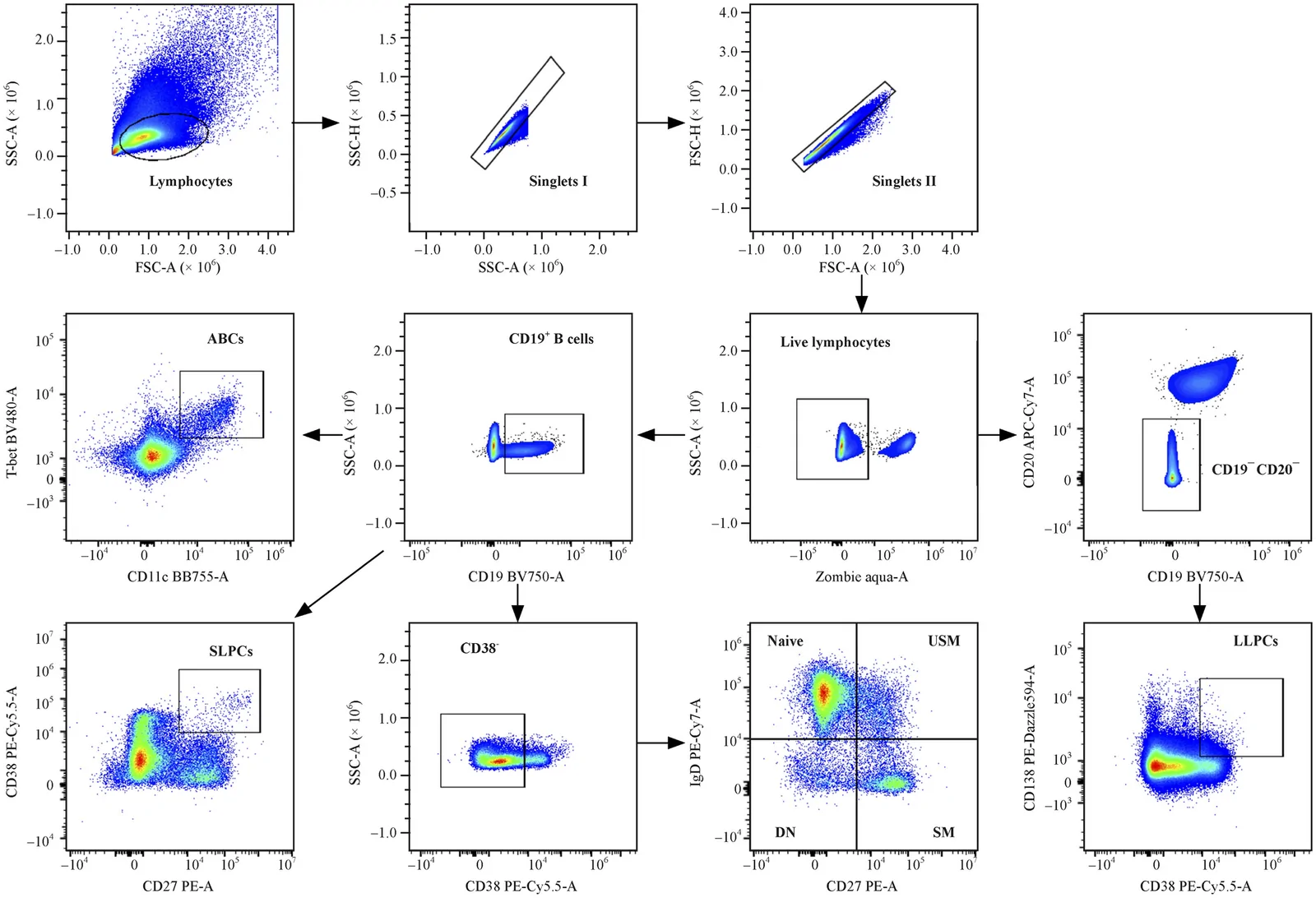
Gating strategy for peripheral B cell subsets. Representative multi-panel flow cytometry plots illustrating the hierarchical gating scheme used to define peripheral B-cell subpopulations. Following initial identification of lymphocytes via FSC-A vs. SSC-A and consecutive doublet exclusion (using SSC-H vs. SSC-A and FSC-H vs. FSC-A), viable cells were isolated via Zombie Aqua™ staining. Plasmablasts (SLPCs: CD27⁺CD38⁺) and ABCs (CD11c⁺T-bet⁺) were gated from CD19⁺ cells, while LLPCs (CD138⁺CD38⁺) were defined within the CD19⁻CD20⁻ population. CD19⁺CD38⁻ B cells were further subdivided into naïve (CD27⁻IgD⁺), USM (CD27⁺IgD⁺), SM (CD27⁺IgD⁻), and DN (CD27⁻IgD⁻) subsets. Data are expressed as frequencies of CD19⁺ B cells or total lymphocytes as indicated. *SSC* side scatter, *FSC* forward scatter, *BB* brilliant blue, *BV* brilliant violet, *A* area, *H* height, *APC* allophycocyanin, *Cy7* cyanine 7, *IgD* immunoglobulin D, *PE* phycoerythrin, *ABCs* age-associated B cells, *SLPCs* short-lived plasma cells, *LLPCs* long-lived plasma cells, *USM* unswitched memory, *SM* switched memory, *DN* double negative

To minimize non-specific antibody binding to Fc receptors, 1.5 × 10^6^ PBMCs were first incubated with Fc receptor block (BioLegend, San Diego, CA, USA) and then stained with a fixable viability dye (BioLegend), followed by surface staining with fluorochrome-conjugated antibodies against CD19, CD20, CD27, IgD, CD38, CD138, and CD11c (BioLegend). Intracellular staining for the transcription factor T-bet (BD Biosciences, San Jose, CA, USA) was performed after fixation and permeabilization using the Foxp3/Transcription Factor Staining Buffer Kit (eBioscience, San Diego, CA, USA). Data acquisition was conducted on a Cytek full-spectrum flow cytometer (Cytek Biosciences, Fremont, CA, USA), and compensation and analysis were performed using FlowJo V10 software. Instrument settings and quality control procedures were standardized across all samples. The gating strategy is illustrated in Fig. 1.

### Statistical analysis

Categorical variables are presented as frequencies and percentages and compared using Fisher’s exact test. Continuous variables were assessed for normality using the Shapiro–Wilk test. Normally distributed data are expressed as mean ± standard deviation and compared using independent-sample *t* tests, whereas non-normally distributed data are expressed as median and interquartile range and compared using the Mann–Whitney *U* test. Spearman’s rank correlation analysis was used to assess the associations between B-cell subsets and clinical or laboratory parameters. To identify factors independently associated with cSLE, multivariate logistic regression analysis was performed, incorporating demographic variables and B-cell subsets. Variables with *P* < 0.10 in univariate analysis were included in the multivariate model. Odds ratios and 95% confidence intervals were reported.

The discriminative performance of individual B-cell subsets and combined models was evaluated using receiver operating characteristic (ROC) curve analysis. Area under the curve was calculated and optimal cutoff values were determined using the Youden index. Combined predictive models were constructed using multivariate logistic regression and evaluated by ROC analysis. All statistical tests were two-tailed, and *P* < 0.05 was considered statistically significant. Statistical analyses were performed using GraphPad Prism (version 10.0) and R software (version 4.3.0).

## Results

### Demographic and clinical characteristics

A total of 64 patients with cSLE and 40 age- and sex-matched HCs were enrolled (Table 1). There were no significant differences in age or sex distribution between the two groups. Among patients with cSLE, the median SLEDAI-2K score was 6 (2.00–11.75), with 53.12% classified as active disease and 46.88% in LLDAS. The majority of patients were seropositive for antinuclear antibodies (ANA) and anti-double-stranded DNA, and nearly half exhibited hypocomplementemia. Most patients were receiving glucocorticoids and/or immunosuppressive therapy at the time of sampling.

**Table 1:** Demographic and clinical characteristics of healthy controls and patients with childhood-onset systemic lupus erythematosus

Characteristics	HCs (*n* = 40)	cSLE (*n* = 64)		*P*
		LLDAS (*n* = 30)	Active (*n* = 34)	
Demographics				
Age (y), mean ± SD	12.28 ± 1.57	12.73 ± 2.24	12.88 ± 1.69	0.108
Sex (female), *n*/*N* (%)	36/40 (90.00)	25/30 (83.33)	29/34 (85.29)	> 0.999
Clinical parameters				
SLEDAI-2K score, median (IQR)	–	2.00 (0.00–2.00)	11.00 (8.00–14.00)	–
Laboratory				
ANA positive, *n*/*N* (%)	–	19/30 (63.33)	31/34 (91.18)	–
Anti-dsDNA positive, *n*/*N* (%)	–	14/30 (46.67)	26/34 (76.47)	–
Hypocomplementemia, *n*/*N* (%)	–	4/30 (13.33)	27/34 (79.41)	–
Proteinuria (≥ 1 +), *n*/*N* (%)	–	0/30 (0.00)	20/34 (58.82)	–
Treatment at sampling				
Drug-naïve, *n*/*N* (%)	–	0/30 (0.00)	4/34 (11.76)	–
Glucocorticoids use, *n*/*N* (%)	–	29/30 (96.67)	30/34 (88.24)	–
Hydroxychloroquine use, *n*/*N* (%)	–	30/30 (100.00)	30/34 (88.24)	–
Mycophenolate mofetil use, *n*/*N* (%)	–	28/30 (93.33)	20/34 (58.82)	–
Cyclophosphamide use, *n*/*N* (%)	–	0/30 (0.00)	3/34 (8.82)	–
Tacrolimus use, *n*/*N* (%)	–	0/30 (0.00)	4/34 (11.76)	–

*P* values for age and sex represent comparisons between HCs (*n* = 40) and total cSLE patients (*n* = 64), calculated using the independent *t* test for age and Fisher's exact test for sex. *HCs* healthy controls, *cSLE* childhood-onset systemic lupus erythematosus, *LLDAS* lupus low disease activity state, *SLEDAI-2K* systemic lupus erythematosus disease activity index 2000, *ANA* antinuclear antibody, *dsDNA* double-stranded DNA. “–” *P* values for other comparisons are not shown

### Global remodeling of the peripheral B-cell landscape

**Fig. 2:**
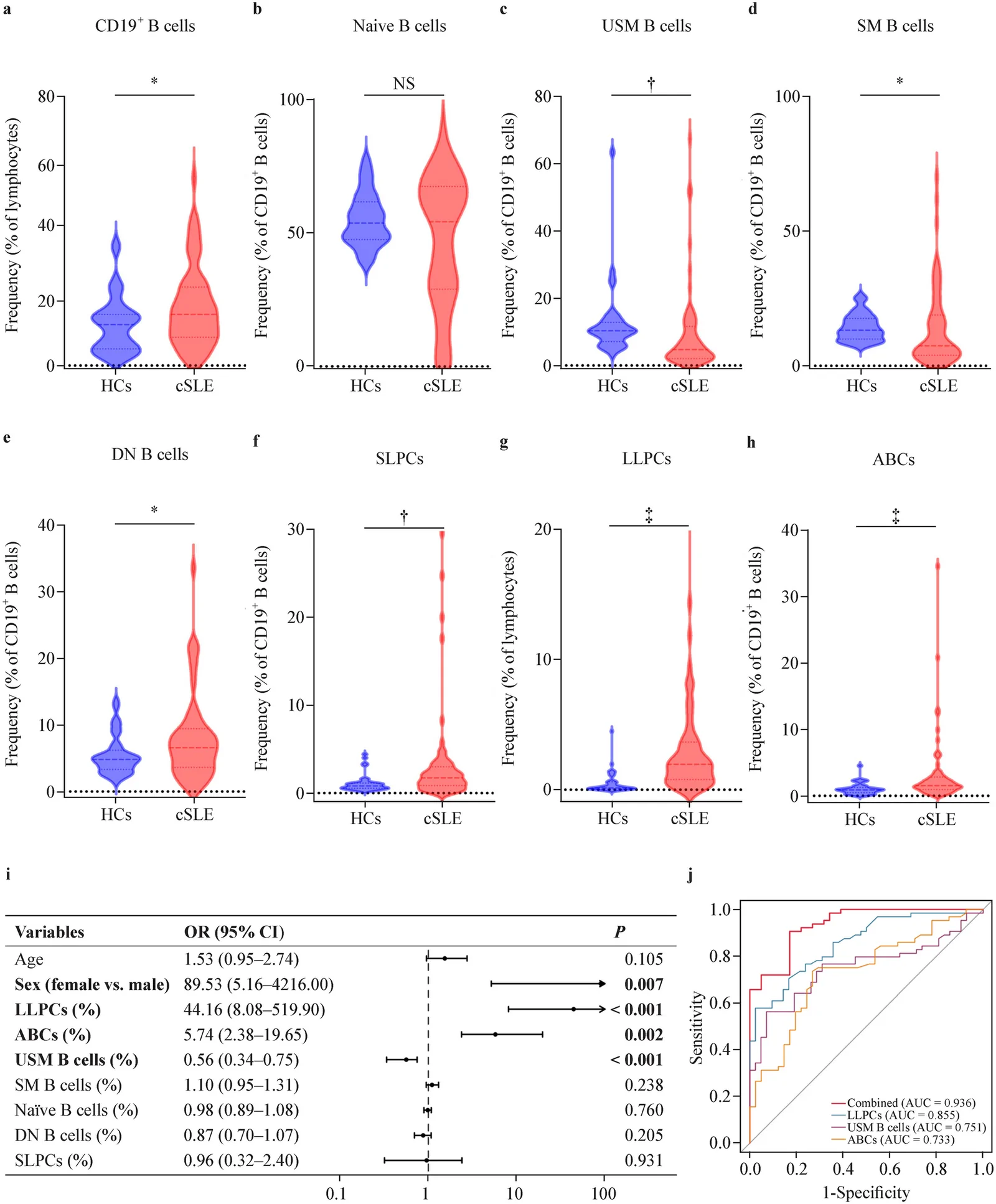
Profiling and diagnostic performance of B-cell subsets in HCs and cSLE patients. Violin plots showing the frequencies of specific B-cell subsets. Data are presented as median and interquartile range (**a–h**). **a** CD19⁺ B cells (% of lymphocytes); **b–e** naïve B cells (CD27⁻IgD⁺), USM B cells (CD27⁺IgD⁺), SM B cells (CD27⁺IgD⁻), and DN B cells (CD27⁻IgD⁻) expressed as percentages of total CD19⁺ B cells; **f** SLPCs; **g** LLPCs; **h** ABCs; **i** forest plot of multivariate logistic regression analysis for variables associated with cSLE. ORs with 95% CIs are shown; **j** receiver operating characteristic curves of selected B-cell subsets and their combination for cSLE diagnosis. AUC values are indicated for ABCs, LLPCs, USM B cells, and the combined model. Statistical significance was determined using the Mann–Whitney *U* test (^*^P < 0.05, ^†^*P* < 0.001, ^‡^*P* < 0.0001). *HCs* healthy controls, *cSLE* childhood-onset systemic lupus erythematosus, *USM* unswitched memory, *SM* switched memory, *DN* double negative, *SLPCs* short-lived plasma cells, *LLPCs* long-lived plasma cells, *ABCs* age-associated B cells, *ORs* odds ratios, *CIs* confidence intervals, *AUC* area under the curve, *NS* not significant

Peripheral immunophenotyping revealed marked alterations in the B-cell makeup in cSLE compared with HCs (Table 2, Fig. 2a–h). The proportion of total CD19⁺ B cells in the lymphocyte population was significantly increased in cSLE. Within the B-cell population, memory subsets, including unswitched memory (USM) and switched memory B cells, were significantly reduced, whereas naïve B cells remained unchanged. In contrast, pathogenic and activated subsets were expanded. DN B cells and ABCs increased significantly. Antibody-secreting populations, including SLPCs and LLPCs, were also markedly elevated. Overall, these findings indicate a shift from conventional memory B-cell populations toward activated and antibody-secreting phenotypes in cSLE.

**Table 2:** Distribution of peripheral B-cell subsets in healthy controls and the overall childhood-onset systemic lupus erythematosus cohort

B-cell subsets (%)	HCs (*n* = 40)	cSLE patients (*n* = 64)	*P*
CD19^+^ B cells	12.20 (4.96–15.25)	15.25 (8.40–23.35)	0.023^*^
Naïve B cells	53.75 (47.63–61.73)	54.25 (29.03–67.45)	0.425
USM B cells	12.24 (7.14–12.88)	9.29 (2.05–11.63)	< 0.001^†^
SM B cells	14.35 (10.00–17.65)	14.18 (3.89–18.88)	0.017^*^
DN B cells	5.42 (3.37–6.25)	7.86 (3.67–9.50)	0.049^*^
SLPCs	0.81 (0.55–1.16)	1.74 (0.86–3.02)	< 0.001^†^
ABCs	0.87 (0.47–4.53)	1.54 (0.96–2.83)	< 0.001^†^
LLPCs	0.50 (0.07–0.63)	3.13 (0.80–3.68)	< 0.001^†^

The cSLE cohort included all enrolled patients (*n* = 64), comprising 30 patients in lupus low disease activity state and 34 patients with active disease. Data are expressed as median (interquartile range). *HCs* healthy controls, *cSLE* childhood-onset systemic lupus erythematosus, *USM* unswitched memory, *SM* switched memory, *DN* double negative, *SLPCs* short-lived plasma cells, *ABCs* age-associated B cells, *LLPCs* long-lived plasma cells. ^*^P < 0.05, ^†^*P* < 0.001

### B-cell signatures associated with cSLE and their diagnostic performance

To identify B-cell subsets independently associated with cSLE, multivariate logistic regression analysis was performed (Fig. 2i). LLPCs and ABCs were positively associated with disease. For USM B cells, there was an inverse association. Among these, LLPCs showed the strongest association with cSLE. ROC analysis demonstrated that LLPCs had the highest discriminative ability among individual subsets, followed by USM B cells and ABCs (Fig. 2j, Table 3). A combined model integrating these subsets substantially improved diagnostic performance, achieving excellent discrimination between cSLE and HCs.

**Table 3:** Receiver operating characteristic analysis of B‑cell subsets for childhood-onset systemic lupus erythematosus identification

Variables	AUC	95% CI	Youden index	Cutoff	Sensitivity	Specificity	*P*
ABCs, %	0.733	0.64–0.83	0.47	1.21	0.73	0.73	< 0.001^*^
LLPCs, %	0.855	0.80–0.93	0.58	1.46	0.58	1.00	< 0.001^*^
USM B cells, %	0.751	0.66–0.85	0.49	5.17	0.56	0.93	< 0.001^*^
Combined model	0.936	0.92–0.99	0.78	0.59	0.88	0.90	< 0.001^*^

The combined model was constructed using logistic regression analysis including ABCs, LLPCs, and USM B cells. *ABCs* age-associated B cells, *LLPCs* long-lived plasma cells, *USM* unswitched memory, *AUC* area under the receiver operating characteristic curve, *CI* confidence interval. ^*^P < 0.001 indicates the area under the curve is significantly different from the horizontal line (AUC = 0.5)

### Distinct B-cell immunophenotypes associated with disease activity and clinical flare

**Fig. 3:**
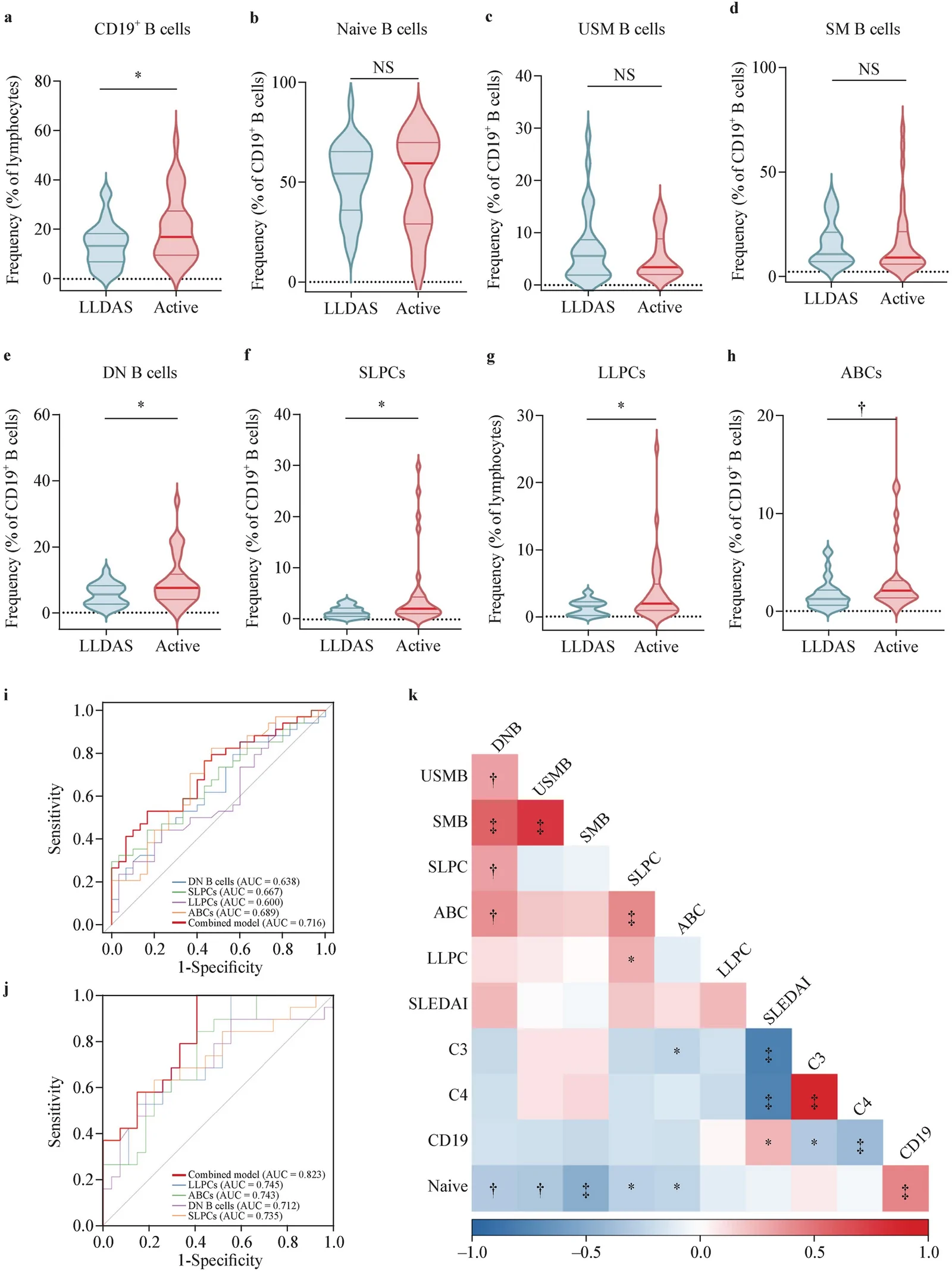
B-cell subset variation and diagnostic performance according to systemic lupus erythematosus disease activity. Violin plots showing frequencies of B-cell subsets in patients with LLDAS and active disease. Data are presented as median and interquartile range (**a–h**). Frequencies of CD19⁺ B cells (**a**) and LLPCs (**g**) are expressed as percentages of total lymphocytes. Frequencies of all other subsets are expressed as percentages of total CD19⁺ B cells, including naïve (**b**), USM (**c**), SM (**d**), DN (**e**), SLPCs (**f**), and ABCs (**h**). Receiver operating characteristic curves for individual subsets and the combined model for discriminating disease activity (**i**, **j**). Spearman correlation heatmap illustrating the relationship between B-cell subsets and clinical parameters (SLEDAI-2K, C3, C4) (**k**). Blue and red colors indicate negative and positive correlations, respectively. Statistical significance was determined using the Mann–Whitney *U* test (^*^P < 0.05, ^†^*P* < 0.01, ^‡^*P* < 0.001). *LLDAS* lupus low disease activity state, *USM* unswitched memory, *SM* switched memory, *DN* double negative, *SLPCs* short-lived plasma cells, *ABCs* age-associated B cells, *LLPCs* long-lived plasma cells, *USMB* unswitched memory B cells, *SMB* switched memory B cells, *SLEDAI-2K* systemic lupus erythematosus disease activity index 2000

We next evaluated B-cell subset changes across disease activity states (Fig. 3a–h, Table 4). Compared with patients in LLDAS, those with active disease were characterized by expansion of activated and antibody-secreting subsets, including DN B cells, ABCs, SLPCs, and LLPCs, accompanied by a modest increase in total CD19⁺ B cells. In contrast, naïve and memory B-cell subsets did not differ significantly, suggesting that disease activity is primarily driven by selective enrichment of pathogenic B-cell populations rather than global shifts across all differentiation stages.

**Table 4:** B-cell subset distribution in patients with childhood-onset systemic lupus erythematosus as a function of disease activity

B-cell subsets (%)	LLDAS (*n* = 30)	Active (*n* = 34)	*P*
CD19^+^ B cells	13.40 (7.28–18.00)	17.05 (10.18–25.85)	0.049^*^
Naïve B cells	54.25 (37.97–64.38)	59.45 (29.68–69.47)	0.950
USM B cells	5.59 (1.99–8.21)	3.44 (2.04–8.21)	0.642
SM B cells	8.71 (5.13–18.50)	7.00 (3.81–18.38)	0.532
DN B cells	5.56 (2.78–8.11)	7.46 (4.28–11.18)	0.034^*^
SLPCs	1.21 (0.62–2.15)	2.10 (1.15–4.16)	0.022^*^
LLPCs	1.54 (0.22–2.12)	1.94 (1.02–4.33)	0.023^*^
ABCs	1.25 (0.69–2.14)	2.11 (1.39–3.00)	0.010^*^

Data are expressed as median (interquartile range). *LLDAS* lupus low disease activity state, *USM* unswitched memory, *SM* switched memory, *DN* double negative, *SLPCs* short-lived plasma cells, *LLPCs* long-lived plasma cells, *ABCs* age-associated B cells. ^*^P < 0.05 indicates a significant difference between the LLDAS and active groups, calculated using the Mann–Whitney *U* test

Importantly, comparison between LLDAS and HCs showed that some abnormalities persisted despite clinical quiescence. This was particularly the case with reduced USM B cells and elevated LLPCs (Supplementary Table 1), indicating incomplete restoration of B-cell homeostasis. The ROC analysis demonstrated that individual subsets showed limited ability to distinguish active disease from LLDAS, whereas a combined model integrating multiple subsets improved discrimination, highlighting the value of coordinated B-cell profiling (Fig. 3i).

In patients with clinical flare, total CD19⁺ B-cell proportions were comparable to those in LLDAS, indicating that relapse is not driven by global B-cell expansion. Instead, flare was characterized by selective enrichment of pathogenic subsets, including ABCs, DN B cells, SLPCs, and LLPCs (Table 5). A combined model incorporating these subsets further improved the identification of flare status (Fig. 3j), outperforming individual markers (Supplementary Table 2).

**Table 5:** B-cell subset distribution in patients with childhood-onset systemic lupus erythematosus as a function of clinical flare status

B-cell subsets (%)	LLDAS (*n* = 30)	Flare (*n* = 19)	*P*
CD19^+^ B cells	13.70 (8.08–17.90)	12.00 (8.37–25.40)	0.518
SLPCs	1.25 (0.64–2.15)	2.53 (1.34–4.53)	0.007^†^
Naïve B cells	54.80 (43.15–64.15)	52.50 (30.80–66.85)	0.616
USM B cells	5.12 (1.90–7.56)	4.08 (2.01–7.93)	1.000
SM B cells	8.28 (4.95–17.20)	6.39 (4.03–15.15)	0.696
DN B cells	4.60 (2.60–7.74)	8.29 (5.24–11.70)	0.015^*^
LLPCs	1.55 (0.18–2.20)	2.35 (0.62–4.03)	0.030^*^
ABCs	1.25 (0.62–2.18)	2.49 (1.53–6.48)	0.002^†^

Data are expressed as median (interquartile range). *LLDAS* lupus low disease activity state, *SLPCs* short-lived plasma cells, *USM* unswitched memory, *SM* switched memory, *DN* double negative, *LLPCs* long-lived plasma cells, *ABCs* age-associated B cells. ^*^P < 0.05 and ^†^*P* < 0.01 indicate significant differences between the LLDAS and flare groups, calculated using the Mann–Whitney *U* test. Clinical flare was defined based on physician assessment and standardized criteria, including worsening clinical manifestations and/or escalation of immunosuppressive therapy

### Correlation between B-cell subsets and clinical parameters

Correlation analysis showed that total CD19⁺ B-cell frequency was positively associated with SLEDAI-2K and inversely correlated with complement levels (Fig. 3k). Among subsets, ABCs exhibited a significant negative correlation with C3 (Supplementary Fig. 1). Other B-cell subsets showed similar trends but they did not reach statistical significance. The subgroup analyses revealed minimal variation in B-cell subsets across different serological and clinical features. Although LLPCs were modestly increased in ANA-positive patients and total CD19⁺ B cells were higher in those with proteinuria, most subsets did not differ significantly (Supplementary Tables 3–5). These findings suggest that B-cell dysregulation represents a systemic feature of cSLE rather than being confined to specific clinical manifestations.

## Discussion

Autoreactive B cells are central drivers of immune dysregulation in cSLE [20]. In this study, we provide a comprehensive characterization of peripheral B-cell remodeling across distinct clinical states and identify that cSLE is associated with marked alterations in B-cell composition. Compared with HCs, cSLE patients exhibited a profound disruption of B-cell homeostasis, characterized by contraction of conventional memory B cells and expansion of atypical and effector subsets, including DN B cells, ABCs, and ASCs. These findings indicate a shift from classical memory pathways toward activated and effector B-cell responses, extending observations from adult SLE into the pediatric context with a more refined subset-level resolution.

B-cell development begins in the bone marrow, progresses through immature and transitional stages into the peripheral circulation, and undergoes differentiation through GC or EF pathways upon antigen-driven activation [21]. Our data demonstrate that distinct clinical states of cSLE exhibit divergent B-cell signatures. Active disease was characterized by broad expansion of activated B-cell subsets and ASCs, whereas clinical flare—despite being embedded within the active disease spectrum—was characterized not by a generalized increase in the overall B-cell compartment but by selective enrichment of pathogenic subsets, particularly ABCs and LLPCs. These observations suggest that differences between disease states are not merely quantitative but also involve qualitative alterations in B-cell compartment organization. Based on these cross-sectional findings, we propose a conceptual framework in which broad activation of B-cell populations and preferential enrichment of ABC and plasma-cell populations represent distinct immunophenotypic features associated with disease activity and flare.

Among the subsets identified, LLPCs are the most strongly associated with clinical disease status and showed the highest discriminative performance. LLPCs are terminally differentiated antibody-producing cells that reside in specialized survival niches and sustain autoantibody production independently of ongoing immune activation [22]. Their persistence in patients achieving LLDAS suggests that clinical remission does not equate to immunological resolution. In contrast to SLPCs, which reflect transient immune activation, LLPCs likely constitute a stable and therapy-resistant compartment that sustains chronic autoimmunity [11]. This finding highlights LLPC persistence as a potential immunological substrate underlying disease chronicity.

Our findings are consistent with emerging evidence that dysregulated EF immune responses represent a central feature of cSLE. Baxter et al. demonstrated that treatment-naïve cSLE patients exhibit expansion of multiple EF-associated populations, including DN2 B cells, anergic B-cell subsets, plasmablasts, and peripheral T helper cells, and showed that this EF signature correlates with disease activity in childhood-onset lupus nephritis [23]. Similarly, Wahadat et al. identified distinct biological phenotypes in cSLE using an integrated multiomic approach and found that activated B-cell populations, particularly CD11c⁺ B cells and plasmablasts, were closely associated with disease activity states rather than specific patterns of organ involvement [24]. Together, these studies suggest a pathogenic model in which aberrant EF-associated B-cell activation drives the generation of autoreactive ASCs and sustains systemic immune activation.

In agreement with these observations, our data demonstrate expansion of both ABCs and DN B cells across active disease states. ABCs, typically characterized by CD11c and T-bet expression, arise under chronic inflammatory conditions and can rapidly differentiate into ASCs via EF pathways, bypassing GC checkpoints [9, 25]. Consistent with their proposed pathogenic role, ABC expansion in our cohort was associated with complement consumption, supporting their link to ongoing immune activation.

Although total DN B cells were significantly increased in cSLE and further enriched during active disease and relapse compared with LLDAS, the DN compartment is increasingly recognized as highly heterogeneous. Functionally distinct DN subsets, including DN1, DN2, and DN3 cells, have been described, with some populations showing stronger links to EF activation and autoimmunity [10]. The lack of CXCR5 and other markers required for comprehensive DN subset characterization limited our ability to distinguish between DN1, DN2, and DN3 populations. Furthermore, studies have further suggested that ABCs themselves may arise through multiple developmental pathways. ABC-like populations have been identified within naïve, memory, and DN B-cell compartments, indicating that ABCs likely comprise a heterogeneous population spanning multiple B-cell compartments rather than a single discrete lineage [25]. Consequently, the observed expansion of total DN B cells in our cohort may reflect accumulation of multiple biologically distinct populations, some of which may overlap phenotypically and functionally with ABCs.

Of note, we observed concurrent enrichment of ABCs and LLPCs during disease flare. Although the biological relationship between these populations cannot be established in this cross-sectional study, their co-expansion is compatible with activation of coordinated effector B-cell programs and warrants further investigation using longitudinal and single-cell approaches. In parallel, we observed a depletion of memory B-cell subsets, particularly USM B cells, which were inversely associated with disease status. This reduction may reflect impaired maintenance of protective humoral memory or preferential skewing toward pathogenic differentiation under inflammatory pressure [26]. Importantly, these alterations persisted even in patients with LLDAS, indicating incomplete restoration of immune homeostasis despite clinical improvement and suggesting a latent vulnerability to relapse.

From a translational perspective, our results highlight the value of integrated B-cell signatures in disease stratification. While individual subsets demonstrated moderate discriminative capacity, combined models incorporating multiple B-cell populations achieved substantially improved performance in distinguishing cSLE from HCs and in identifying disease activity and flare. These findings underscore that coordinated rather than isolated changes across the B-cell compartment more accurately capture the underlying disease biology.

Our findings also have therapeutic implications. Recent advances in childhood-onset lupus nephritis have highlighted a shift toward earlier incorporation of B-cell–targeted therapies, including rituximab, belimumab, and obinutuzumab, as part of efforts to improve disease control and reduce corticosteroid exposure [27]. However, the persistence of LLPCs, which typically lack CD19 and CD20 expression, may limit the efficacy of conventional B-cell–depleting therapies such as rituximab and contribute to incomplete or heterogeneous treatment responses. Targeting the plasma-cell compartment or its survival pathways may therefore be necessary to achieve durable disease control [28–31]. Together, these findings support a therapeutic framework that extends beyond conventional B-cell depletion and incorporates interventions directed at both pathogenic precursor populations and LLPCs.

Several limitations should be acknowledged. First, our cross-sectional design precludes direct assessment of temporal dynamics and causality. Longitudinal studies are required to determine how B-cell immunophenotypes evolve in relation to disease activity and clinical outcomes. Second, the sample size, particularly in the flare subgroup, was relatively limited. Third, functional studies were not performed to directly confirm lineage differentiation relationships or mechanistic interactions among B-cell subsets. Finally, the potential impact of immunosuppressive therapy on B-cell composition cannot be fully excluded.

In conclusion, this study demonstrates that cSLE is characterized by profound remodeling of the B-cell compartment, with distinct B-cell immunophenotypes observed across different disease activity states, particularly involving expansion of ABCs and plasma-cell populations. The persistence of LLPCs during clinical quiescence suggests that abnormalities within the plasma-cell compartment may remain detectable despite achievement of low disease activity. Future longitudinal and mechanistic studies are warranted to determine the clinical significance of these persistent alterations and their potential value as therapeutic targets in cSLE.

## Supplementary Information

Below is the link to the electronic supplementary material.

Supplementary file1 (PDF 388 KB)

## Data Availability

Datasets generated and/or analyzed during the current study are available from the corresponding author on reasonable request.
