# Supplementary material for: Distinct peripheral B-cell immunophenotypes define clinical heterogeneity in childhood-onset systemic lupus erythematosus
Source: World J Pediatr. 2026 Sep 1;22(8):910–21. doi: 10.1007/s12519-026-01082-x (PMC13615152; doi:10.1007/s12519-026-01082-x)
Supplement: Supplementary file 1 [file 12519_2026_1082_MOESM1_ESM.pdf]

Supplementary Table 1 Distribution of B-cell subsets in HCs and patients with LLDAS

B-cell subsets	HCs (n = 40)	LLDAS (n = 30)	P
$CD19^+$ B cells	12.20 (5.27–14.95)	13.40 (7.28–18.00)	0.489
Naïve B cells	53.75 (47.68–61.58)	54.25 (37.97–64.38)	0.877
USM B cells	10.40 (7.58–12.83)	5.59 (1.99–8.21)	0.002^*
SM B cells	13.30 (10.07–17.35)	8.71 (5.13–18.50)	0.202
DN B cells	4.86 (3.62–5.86)	5.56 (2.78–8.11)	0.990
SLPCs	0.81 (0.56–1.13)	1.21 (0.62–2.15)	0.154
LLPCs	0.14 (0.07–0.60)	1.54 (0.22–2.12)	0.001^*
ABCs	0.87 (0.48–1.21)	1.25 (0.69–2.14)	0.076

$CD19^+$ B cells and LLPCs are expressed as a percentage of total lymphocytes, while other B-cell subsets are expressed as a percentage of total $CD19^+$ B cells. Data are expressed as median (interquartile range); $^*P < 0.01$ indicates a significant difference between the HCs and LLDAS groups, calculated using the Mann–Whitney U test. *HCs* healthy controls, *LLDAS* lupus low disease activity state, *USM* unswitched memory, *SM* switched memory, *DN* double negative, *SLPCs* short-lived plasma cells, *LLPCs* long-lived plasma cells, *ABCs* age-associated B cells

Supplementary Table 2 Diagnostic performance of B-cell subsets in predicting clinical flares in cSLE

Variables	AUC	95% CI	Youden index	Cutoff	Sensitivity	Specificity	P
LLPCs, %	0.75	0.60–0.89	0.44	0.87	1.00	0.44	$0.005^\dagger$
ABCs, %	0.74	0.60–0.89	0.44	1.51	0.84	0.59	$0.005^\dagger$
DN B cells, %	0.71	0.55–0.87	0.39	8.17	0.58	0.82	0.015^*
SLPCs, %	0.74	0.58–0.89	0.43	2.40	0.58	0.85	$0.007^\dagger$
Combined model	0.82	0.70–0.94	0.59	0.24	1.00	0.59	$< 0.001^\dagger$

$CD19^+$ B cells and LLPCs are expressed as a percentage of total lymphocytes, while other B-cell subsets are expressed as a percentage of total $CD19^+$ B cells. AUC values with 95% CIs are presented. $^*P < 0.05$ and $^\dagger P < 0.01$ indicates statistical significance. The combined model was constructed using multivariate logistic regression analysis. *cSLE* childhood-onset systemic lupus erythematosus, *LLPCs* long-lived plasma cells, *ABCs* age-associated B cells, *DN* double negative, *SLPCs* short-lived plasma cells, *AUC* area under the curve, *CI* confidence interval

Supplementary Table 3 Distribution of B-cell subsets based on ANA status

B cells subsets (%)	ANA positive (n = 50)	ANA negative (n = 14)	P
$CD19^+$ B cells	5.85 (9.97–24.08)	11.20 (4.86–18.35)	0.066
Naïve B cells	59.10 (33.77–68.28)	48.60 (33.17–62.78)	0.475
USM B cells	3.85 (2.03–8.12)	5.81 (2.16–11.24)	0.480
SM B cells	7.45 (4.34–17.25)	11.17 (5.26–24.10)	0.475
DN B cells	6.86 (3.26–8.84)	5.94 (3.68–10.01)	0.884
SLPCs	1.79 (0.74–2.94)	1.45 (0.94–2.87)	0.661
LLPCs	1.85 (0.74–3.68)	0.81 (0.17–2.12)	0.047*
ABCs	1.50 (0.96–2.74)	2.12 (1.38–3.22)	0.350

$CD19^+$ B cells and LLPCs are expressed as a percentage of total lymphocytes, while other B-cell subsets are expressed as a percentage of total $CD19^+$ B cells. Data are expressed as median (interquartile range); $^*P< 0.05$ indicates a significant difference between groups, calculated using the Mann–Whitney *U* test. *ANA* antinuclear antibody, *USM* unswitched memory, *SM* switched memory, *DN* double negative, *SLPCs* short-lived plasma cells, *LLPCs* long-lived plasma cells, *ABCs* age-associated B cells

Supplementary Table 4. Distribution of B-cell subsets based on dsDNA status

B cells subsets (%)	dsDNA positive (n = 40)	dsDNA negative (n = 24)	P
$CD19^+$ B cells	16.95 (8.47–24.73)	13.40 (7.70–17.68)	0.095
Naïve B cells	58.30 (32.23–69.83)	52.15 (36.02–63.78)	0.501
USM B cells	3.01 (2.02–7.66)	5.54 (3.14–9.24)	0.289
SM B cells	7.00 (3.76–19.73)	13.15 (5.74–17.55)	0.360
DN B cells	6.26 (3.55–9.22)	6.81 (3.56–8.35)	0.830
SLPCs	1.59 (0.67–3.28)	1.94 (1.01–2.92)	0.912
LLPCs	1.84 (0.65–3.23)	1.73 (0.47–3.34)	0.729
ABCs	1.50 (0.84–2.31)	2.10 (1.30–3.57)	0.142

$CD19^+$ B cells and LLPCs are expressed as a percentage of total lymphocytes, while other B-cell subsets are expressed as a percentage of total $CD19^+$ B cells. Data are expressed as median (interquartile range); $^*P < 0.05$ indicates a significant difference between groups, calculated using the Mann–Whitney *U* test. *dsDNA* double-stranded DNA, *USM* unswitched memory, *SM* switched memory, *DN* double negative, *SLPCs* short-lived plasma cells, *LLPCs* long-lived plasma cells, *ABCs* age-associated B cells

Supplementary Table 5 Distribution of B-cell subsets in cSLE with and without proteinuria

B cells subsets (%)	Proteinuria (*n* = 20)	Non-proteinuria (*n* = 44)	*P*
$CD19^+$ B cells	22.05 (11.70–34.80)	13.40 (8.28–18.42)	0.029*
Naïve B cells	51.10 (27.58–69.75)	56.10 (37.88–67.35)	0.553
USM B cells	3.56 (2.09–10.09)	4.77 (2.00–7.89)	0.988
SM B cells	7.00 (2.61–26.93)	8.71 (5.01–17.68)	0.817
DN B cells	7.05 (3.72–11.60)	5.94 (3.56–8.41)	0.491
SLPCs	2.08 (1.20–3.58)	1.43 (0.65–2.62)	0.145
LLPCs	2.35 (1.08–5.03)	1.62 (0.51–2.57)	0.111
ABCs	1.54 (1.27–2.51)	1.62 (0.87–3.00)	0.925

$CD19^+$ B cells and LLPCs are expressed as a percentage of total lymphocytes, while other B-cell subsets are expressed as a percentage of total $CD19^+$ B cells. Data are expressed as median (interquartile range); $^*P < 0.05$ indicates a significant difference between groups, calculated using the Mann–Whitney *U* test. *USM* unswitched memory, *SM* switched memory, *DN* double negative, *SLPCs* short-lived plasma cells, *LLPCs* long-lived plasma cells, *ABCs* age-associated B cells

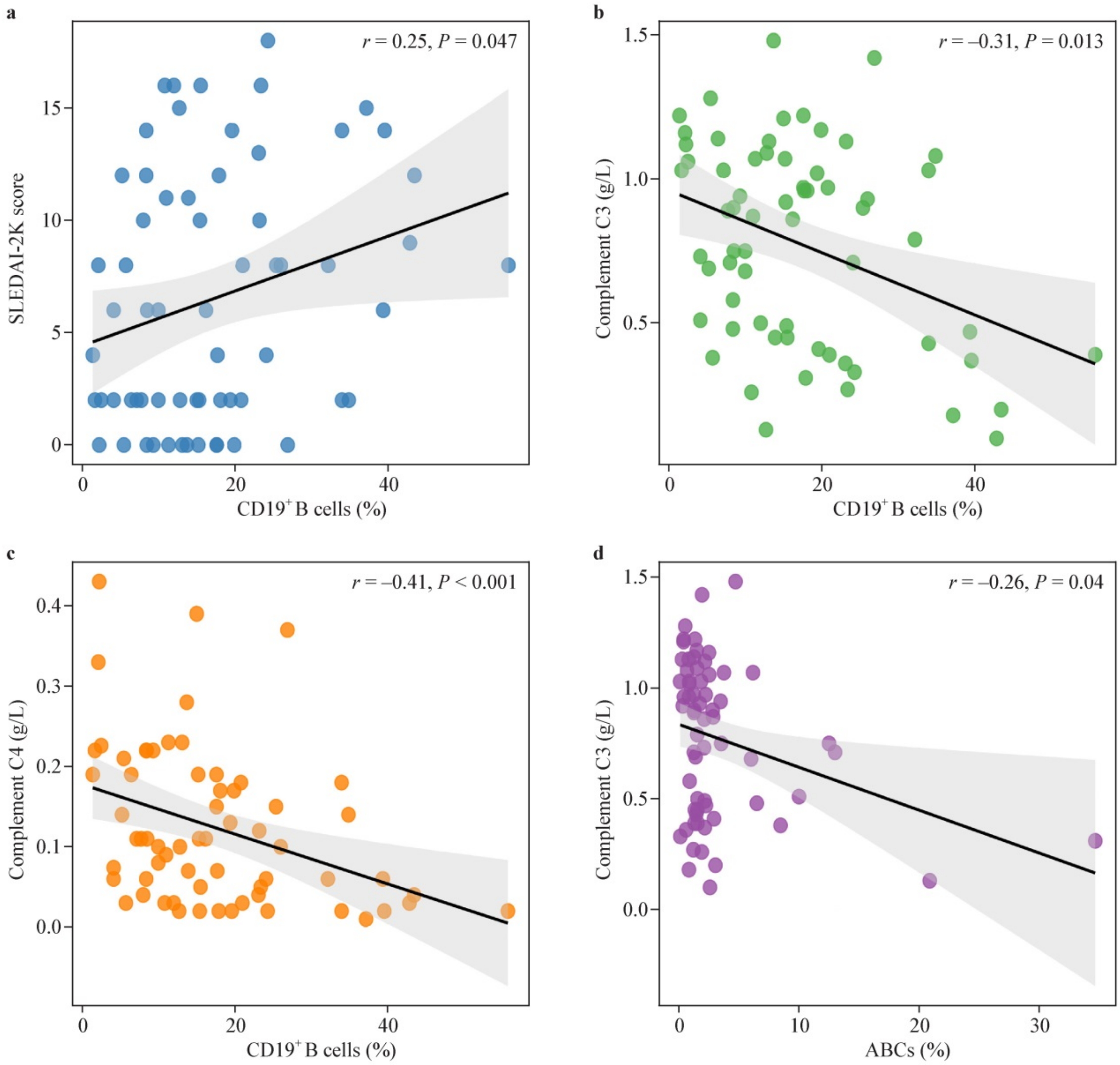

Supplementary Fig. 1 Spearman correlations between B-cell subsets and clinical indicators in cSLE patients. Scatter plots with linear regression fits and 95% confidence intervals (shaded areas) illustrating the relationships between specific B-cell subsets and clinical markers. **a–c** Frequencies of $CD19^+$ B cells show a positive correlation with SLEDAI-2K scores (**a**; $r = 0.25$, $P = 0.047$) and significant negative correlations with complement C3 (**b**; $r = -0.31$, $P = 0.013$) and C4 levels (**c**; $r = -0.41$, $P < 0.001$); **d** ABCs frequencies exhibit a negative correlation with complement C3 levels ($r = -0.26$, $P = 0.04$). $P < 0.05$ was considered statistically significant. *cSLE* childhood-onset systemic lupus erythematosus, *ABCs* age-associated B cells, *SLEDAI-2K* systemic lupus erythematosus disease activity index 2000
